# Chickpea aquafaba: a systematic review of the different processes for obtaining and their nutritional and technological characteristics

**DOI:** 10.1007/s13197-023-05920-y

**Published:** 2024-01-26

**Authors:** Bianca de Barros Miranda, Graziella Silva Holanda, António Raposo, Dayanne da Costa Maynard, Raquel Braz Assunção Botelho, Bernardo Romão, Viviani Ruffo de Oliveira, Renata Puppin Zandonadi

**Affiliations:** 1 Department of Nutrition, University of Brasilia, Campus Universitário Darcy Ribeiro, Brasília, Distrito Federal 70910-900 Brazil; 2 CBIOS (Research Center for Biosciences and Health Technologies), Universidade Lusófona de Humanidades e Tecnologias, Campo Grande 376, 1749-024 Lisboa, Portugal

**Keywords:** Processes for obtaining aquafaba, Chickpea, Chemical composition, Foam stability, Technological properties

## Abstract

Aquafaba is the residual water from cooking chickpea in water. It has a high gelling ability, allowing it to create stable gels. However, those functional properties depend on the legume composition, genotype, cooking time, pressure, and temperature. This study aimed to evaluate the different processes for obtaining aquafaba and compare their nutritional composition and technological characteristics using a systematic review. The authors performed the systematic review by performing specific search strategies for Scopus, Web of Science, Pubmed, Lilacs, Google Scholar, and ProQuest. A total of 17 studies were analyzed. Of them, 17.64% (n = 3) used the wastewater from canned chickpeas, 17.64% (n = 3) compared the wastewater of canned chickpeas and dry grains, and 58.82% (n = 10) used dry chickpeas. Studies used different methods to analyze the protein content. The most used (n = 5) was the Association of Official Analytical Chemists (AOAC). The aquafaba presented carbohydrates at 2.03–2.59 g/100ml; protein at 0.0.8–2.8 g/100ml; and fat at 0.07–0.1 g/100ml. In general, preparing aquafaba followed: soaking (8–10 h at 4 °C—1 chickpea: 4 water), pressure cooking (30 min—2 chickpea: 3 water), and refrigerating (24h/4 °C). In general, the results showed the following steps to prepare aquafaba: soaking for 8–10 h at 4 °C at the proportion of 1:4 (chickpea:water), pressure cooking for 30 min in the proportion of 2:3 (chickpea: water), and refrigerating 24 h/4 °C. These procedures in a homemade aquafaba presented the best results, considering foam development and higher stability. The aquafaba from canned chickpeas has a higher foam-ability and lower emulsion properties than homemade cooking aquafaba.

**Supplementary Information:**

The online version contains supplementary material available at 10.1007/s13197-023-05920-y.

## Introduction

Plant-based food products as substitutes for animal sources have been considered healthy and eco-friendly in the past few years. This market growth is mainly from populations with specific dietary choices, such as vegans and vegetarians (He et al. 2019). The demand for alternative egg products has increased, especially for those that do not compromise the sensory, mainly taste and texture, and technological properties such as foaming, emulsifying, and heat coagulation that the eggs contribute to the food (Buhl et al. 2019a; Meurer 2019). Plant-based products replicating eggs’ qualities are becoming increasingly popular among vegetarian individuals and people allergic to animal food. Among food allergies, eggs (mainly egg whites) are one of the most common, particularly among children, with prevalences ranging from 0.5 to 2% (Caubet and Wang 2011; Mustafa et al. 2018; Shim et al. 2018). For those reasons, the search for products using egg substitutes increases without giving up the taste and functional properties that eggs bring to food (Buhl et al. 2019a).

Aquafaba, the residual byproduct solution (about 90–95% water) from canning, boiling seeds, or other pulses in water, may improve the sensory and technological quality of egg-free food products due to its emulsion, foamability, stability, moisture retention, adhesion, gelation, and thickening properties. The most common one is produced using chickpeas. Chickpeas are grown mainly in South Asia, accounting for around three-fourths of the world’s area (planted on a surface of 11 million ha). Its sales in the global market are projected to increase from US$ 12 million to U$ 21 million in 2032, highlighting the importance of aquafaba in the world (Future Market Insights 2022). However, only recently was aquafaba understood as an ingredient with technological importance, once interpreted as a waste of no industrial importance whatsoever (Mustafa and Reaney 2020). In this manner, multiple studies report the use of chickpea aquafaba as an enhancer or substitute to mostly egg whites, providing a viable usage and adding monetary value to an ingredient previously wasted (Mustafa et al. 2018; Anwar et al. 2019; Buhl et al. 2019a, 2019b; Raikos et al. 2020; Nguyen et al. 2021). The properties of chickpea aquafaba are mainly due to its proteins, carbohydrates (starch, oligosaccharide, cellulose, hemicellulose, lignin), polysaccharide-protein complexes, saponins, and phenolic compounds (Alsalman et al. 2020a, 2020b; Alsalman and Ramaswamy 2021a; He et al. 2021).

Aquafaba’s use in food products depends on its consistency, composition, and quality, and its production standardization is a difficult task necessary to ensure the products’ quality. Several parameters to assure its composition and functionality should be considered in aquafaba production, such as the type of pulse, water/pulse ratio, temperature, cooking pressure, and cooking time. Some studies evaluated aquafaba production or composition (Shim et al. 2018; Buhl et al. 2019a; He 2019; He et al. 2019; Lafarga et al. 2019a; Meurer 2019; Nguyệt 2019; Alsalman 2020; Alsalman et al. 2020b; Alsalman and Ramaswamy 2021a, 2021b; Aslan and Ertaş 2021; Editors et al. 2021; Landert et al. 2021; Nguyen et al. 2021). However, to our knowledge, there is no production standardization, and the nutritional and technological properties of aquafaba have not yet been well explored. The hypothesis is that there is the best way to produce chickpea aquafaba, considering nutritional and technological characteristics. Therefore, this study aimed to evaluate the different processes for obtaining chickpea aquafaba and compare their nutritional quality and technological characteristics through a systematic review.

## Methods

This systematic review was performed following the Preferred Reporting Items for Systematic Reviews and Meta-Analyses (PRISMA) and its Checklist (Moher et al. 2009; Page et al. 2021). Also, registers of the ongoing systematic reviews were searched via PROSPERO (Centre for Reviews and Dissemination). The protocol was executed according to the following steps:

### Inclusion and exclusion criteria

The inclusion criteria were studies evaluating the properties of chickpea aquafaba (technological and nutritional) with no limitations in terms of language or time. The exclusion criteria applied were: (1) reviews, letters, conference abstracts, case reports, books, clinical studies, and review studies; (2) studies that did not evaluate the properties of aquafaba but tried to include it in the formulation of a food product; (3) studies that focused on the improvements of aquafaba through treatments; (4) studies evaluating aquafaba made from other pulses that not chickpeas (e.g., peas, pigeon beans); The excluded studies, and their reasons were inserted as a supplementary file (Table S1).

### Information source

Five electronic databases were searched in February 2022: Medline, Embase, Lilacs, PubMed, and Web of Science, complemented by gray literature research in Google Scholar and ProQuest. The reference lists of the selected papers were checked, as relevant studies may have been missed during the data search.

### Search strategy

The appropriate combinations of truncation and keywords were selected and adapted for searching each database. The software Rayyan® (Qatar Computing Research Institute‐QCRI) was used to aid in the selection and deletion of duplicate articles. The Mendeley desktop software was used to manage all the references (Table S2—Indexers used to select publications that jointly or separately address words related to aquafaba and its properties).

### Studies selection and data collection

There were two phases to the study selection process. In phase one, all identified references in the databases had their titles and abstracts reviewed separately by two reviewers (B.B.M, G.S.H). The items that did not match the eligibility criteria were discarded. In phase two, the entire texts of the selected articles were subjected to the eligibility criteria by the same reviewers (B.B.M, G.S.H). In cases of conflict, regardless of the phase, the topic was debated until the two reviewers agreed. In circumstances where there was no agreement, the final judgment was made by a third reviewer (D.C.M). The final decision was always performed after reading the full papers.

**Fig. 1:**
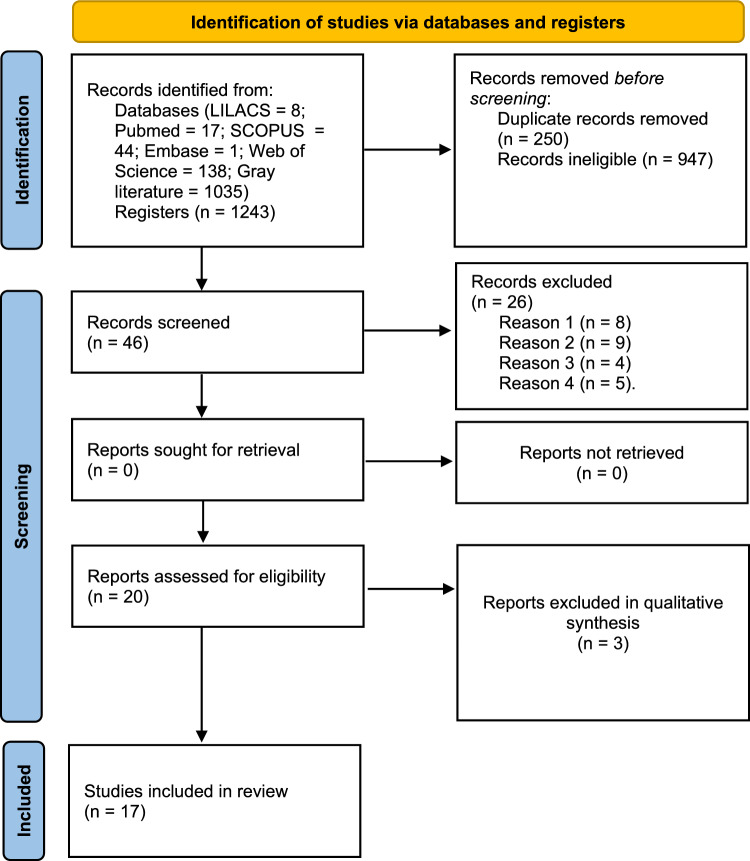
Systematic review flowchart adapted from PRISMA. Exclusion criteria: REASON 1—comments, letters, conference, review, abstracts, papers, and books (n = 8); REASON 2—studies that do not evaluate the properties of aquafaba, but try to include it in the formulation of a product (n = 9); REASON 3—it does not evaluate the properties, but improves aquafaba through treatments (n = 4); REASON 4—studies evaluating aquafaba from different pulses (n = 5);

The following items were collected in the data collection process: authors and year of publication, research country, the study's objective, the proportion of water and chickpeas, methods, and main results. The report was based on the PRISMA flowchart (Fig. 1).

### Risk of bias (RB)

A particular instrument was constructed for this study to evaluate the Risk of Bias using well-established classical and literature criteria and expert guidance, based on instructions provided by the Joanna Briggs Institute (Aromataris and Munn 2021). Six questions were included in the assessment instrument for the bias risk of the chosen 17 studies: (1) Was the Study design appropriate?; (2) Was the statistical analysis adequate to the objective of the study?; (3) Were objective, standard criteria used for measuring the condition?; (4) Did the results answer the main question?; (5) Were strategies to deal with confounding factors stated?; (6) Were the outcomes measured in a valid and reliable way?. When the study received a score of up to 49% “yes,” the risk of bias was classified as High, and when the study received a score of 50–69% “yes,” the risk of bias was classified as Moderate and Low when the study reached more than 70% yes (Table S3).

## Results

A total of 1243 articles were initially found in the electronic databases. After removing duplicates, 250 articles titles and abstracts were selected and read. After reading the abstracts, 46 studies were selected for full-text reading. No study records were chosen from the references list of full-text articles. After reviewing the papers, 26 articles were excluded: comments, letters, conference, review, abstracts, papers, and books (n = 8); studies that do not evaluate the properties of aquafaba, but try to include it in the formulation of a product (n = 9); it does not evaluate the properties, but improves aquafaba through treatments (n = 4); studies evaluating aquafaba from different pulses (n = 5) (Table S1—Supplementary material). By the end of the process, 17 studies met inclusion criteria and were considered for this systematic review. The flowchart of the study identification, screening, and inclusion process is in Fig. 1.

### Studies characteristics

The studies were carried out in the following countries: Canada (n = 7; 41.17%) (Mustafa et al. 2018; Shim et al. 2018; He 2019; He et al. 2019; Alsalman 2020; Alsalman et al. 2020a; Alsalman and Ramaswamy 2021a), Vietnam (n = 3; 17.64%) (Nguyệt 2019; Editors et al. 2021; Nguyen et al. 2021), Brazil (n = 3; 17.64%) (Meurer 2019; Landert et al. 2021), China (n = 2; 11.76%) (Mustafa et al. 2018; He et al. 2019), USA (n = 1; 5.88%) (Nguyen et al. 2021), Spain (n = 1; 5.88%) (Lafarga et al. 2019a), France (n = 1; 5.88%) (Escadellas et al. 2022), Lebanon (n = 1; 5.88%) (Shim et al. 2018), Turkey (n = 1; 5.88%) (Aslan and Ertaş 2021), Denmark (n = 1; 5.88%) (Buhl et al. 2019a), Korea (n = 1; 5.88%) (He et al. 2019). The date range for the included studies was between 2009 and 2018 (Table 1).

**Table 1:** Main descriptive characteristics and results from the included studies

Reference, Year, and Country	Objectives	Proportion of chickpea/water	Methods	Results of aquafaba nutritional composition	Results of technological properties
He et al. (2019) Canada, China, and Korea	Prepare aquafaba from a variety of chickpea cultivars and use it to make food oil emulsions, then compare the properties of those emulsions	• Hydrated grain: Yes	Emulsifying stability	No information	Aquafaba emulsion capacity and stability ranged from 1.10 to 1.30 m^2^/g and 71.9 to 77.1%, respectively. Significant correlation between the proximate composition of chickpea (carbohydrate, protein) and emulsion capacity and stability. The lower the chickpeas' carbohydrate content, the lower the emulsion properties
		◦ Proportion (dry grain:water): 1:4	Method used to determine protein content: Association of Official Analytical Chemists (AOAC) methods		
		◦ Was the water discarded: Yes			
		• Cooking:			
		◦ Pressure cooker for 30 min			
		◦ Proportion (hydrated grain:water): 1:1			
Buhl et al. (2019a)	Determine the protein content of aquafaba made from canned chickpeas and test for functional properties in foams and emulsions, as well as the effect of pH and NaCl on these properties	• Wastewater from chickpea canning	Foaming properties, emulsifying activity, and stability	Protein: 1.3%	Foam capacity: Centrifuged aquafaba had a significantly lower foam capacity when compared to egg white. However, it was not affected by changes in the pH level or the
Denmark		◦ The proportion of grain/water was not mentioned		The dry matter content of centrifuged aquafaba (carbohydrates in the form of sugars, water-soluble and insoluble fiber, and protein): 7.89 ± 0.09% w/v of the herbal liquid of aquafaba	NaCl within the studied range. The foam produced by the centrifuged aquafaba was
			Method used to determine protein content: BCA, Thermo Scientific™, USA		more moisture than the foam produced by the egg white, as it had a higher proportion of liquid. In addition, the protein surface charge affects foam stability
					Emulsion properties: the centrifugated aquafaba-based emulsions showed a significantly higher emulsifying activity index and stability index than emulsions prepared by egg white. The emulsifying activity and stability index were not affected by changes in NaCl. However, the change in pH affected the diameter of the mean particles
Aslan and Ertaş (2021) Turkey	Optimize the variables of the aquafaba foam in the drying process	• Hydrated grain: No	Foaming properties	No information	The optimum temperature for the foam-mat drying of aquafaba liquid was 70 °C
		• Cooking:			The optimum formulation was determined as 0.716% carboxymethylcellulose, 0.165% Na-alginate, and 0.119% polydextrose for foaming properties
		◦ Boiling water for 30 min			
		◦ Proportion (dry grain:water): 1:5			
Mustafa et al. (2018) Canada and China	(1) Investigate foaming and emulsifying capabilities for aquafaba made from commercially available chickpeas. (2) Choose aquafaba with the best functional properties to replace egg white in a sponge cake recipe	• Wastewater from chickpea canning	Foaming capacity and stability, emulsifying capacity and stability	No information	The tested commercial brands have different foaming and emulsifying capabilities. Foaming capacity and stability ranged from 182 to 476% and 77 to 92%, respectively, with emulsion stability varying from 60 to 80%
	(3) Compare physiochemical and textural properties of aquafaba and egg white in cake	◦ The proportion of grain/water was not mentioned			
Alsalman et al. (2020a) Canada	Use a statistical sound experimental design to evaluate different ways of cooking chickpeas to obtain aquafaba by varying the chickpeas, the proportion of water (CPCWR), and the cooking time	Wastewater from chickpeas and dried grain	Foaming capacity	Protein content: 0.5–1% (wasterwater)	The optimal conditions were 2:3 chickpea to water ratio cooked for 60 min
		• Wastewater from chickpea canning			
		◦ The proportion of grain/water was not mentioned	Method used to determine protein content: Bradford (1976)		
		• Hydrated grain: Yes			
		◦ Proportion: no information			
		◦ Was the hydration water discarded? no information			
		• Cooking:			
		◦ Pressure cooker			
		◦ Proportion (hydrated grain:water): 1:2, 1:4, and 2:3			
Landert et al. (2021) Brazil	Standardize the process of obtaining homemade aquafaba for application in vegan cooking	Wastewater from chickpeas and dried grain	Foaming capacity and stability	Protein content: 1.7 g/100 ml (wastewater) and 3% (homemade)	Aquafaba from canned chickpeas has a higher foam volume. As for homemade aquafaba, the best result was achieved with a ratio of 2:3
		• Wastewater from chickpea canning			
		◦ The proportion of grain/water was not mentioned	Method used to determine protein content: Association of Official Analytical Chemists (AOAC)		
		• Hydrated grain: Yes			
		◦ Proportion: no information			
		◦ Was the hydration water discarded? Yes			
		• Cooking:			
		◦ Pressure cooker for 20 min			
		◦ Proportion (hydrated grain:water): 2:2; 2:3 and 2:4			
Alsalman and Ramaswamy (2021a) Canada	Investigate the enhancement of gel strength, crystallinity, and starch digestibility of aqueous aquafaba slurry and compare them to those from untreated samples	• Hydrated grain: Yes	Foaming capacity and stability, emulsion capacity, stability, gel strength, crystallinity, and starch digestibility	No information	Foaming capacity and stability: An increase in CWR (chickpea: boiling water ratio) and pH will cause a decrease in foam capacity
		◦ Proportion: no information			
		◦ Was the water discarded? -			Emulsion capacity and stability are higher with lower ph and CWR values
		• Cooking:			
		◦ Pressure cooker for 60 min			
		◦ Proportion (hydrated grain:water): 1.5:3.5			
Lafarga et al. (2019a) Spain	Optimize the pH and domestic boiling conditions (chickpea: water ratio) required to increase the foaming and emulsifying capabilities of CCW (RSM) using response surface methodology	• Hydrated grain: Yes	Foaming capacity and stability, emulsion capacity, and stability	The protein concentration of the aquafaba obtained at CWR of 1:5, 1:3.25, and 1:1.5 was measured as 0.48 ± 0.01, 0.23 ± 0.04, and 0.08 ± 0.00%, respectively	Both the boiling conditions and the pH adjustment were critical when preparing chickpea aquafaba at home to maximize the foaming and emulsifying abilities of the chickpea aquafaba and the stability of the generated foams and emulsions
		◦ Proportion (dry grain:water): 1:3			
		◦ Was the hydration water discarded? Yes			
		• Cooking:			
		◦ Boiling water for 190 min			
		◦ Proportion (hydrated grain:water): 1:3.25; 1:5 and 1:1.5			
Shim et al. (2018) Canada and Líbano	Determine the components of aquafaba that contribute to foaming properties	• Wastewater from chickpea canning	Protein and carbon content, functional properties of aquafaba, foaming capacity, chickpea seed color parameters	Protein content considering wet basis (1.26–1.56%)	The aquafaba from 10 commercial products varied significantly in foam volume and stability. Brands D and H showed greater foam stability after 14 h at storage, and no additives were included other than water and chickpeas
			Method used to determine protein content: Association of Official Analytical Chemists (AOAC) [22]		
Nguyet et al. (2021) (Editors et al. 2021) Vietnam	Use high-pressure processing to improve chickpeas and their byproduct “aquafaba” qualities and functional properties	• Hydrated grain: Yes	Foaming capacity and stability	No information	The ratio of 1:4 is considered to be the most suitable ratio to obtain the highest values of foaming capacity and stability
		◦ Proportion (dry grain:water): 1:4			
		◦ Was the water discarded: no information			
		• Cooking:			
		◦ Boiling water for 45 min			
		◦ Proportion (hydrated grain:water): 1:3, 1:4, and 1:5			
He (2019)	(1) Determine which chickpea cultivar produces aquafaba with the best emulsion properties;	◦ Hydrated grain: Yes	Emulsion turbidity and capacity	No information	Aquafaba prepared by soaking chickpea seed in 4 °C water for 16 h and cooking for 30 min displayed the highest emulsion capacity (1.30 m^2^ g^−1^) and stability (77.1%)
	(2) Determine grain composition and physicochemical properties of the different chickpea cultivars used in this study;	◦ Proportion (dry grain): 1:4			
Canada		◦ Was the hydration water discarded? Yes	Method used to determine protein content:		
	(3) Determine correlations among AQ emulsion properties, chickpea composition, and chickpea physicochemical properties; and	◦ Cooking:	Association of Official Analytical Chemists (AOAC) methods		
		◦ Pressure cooker for 30 min			
	(4) Standardize the conditions for aquafaba preparation and compare the influences of different commercial drying methods on aquafaba emulsion properties	◦ Proportion (hydrated grain:water): 1:1			
Alsalman (2020) Canada	Enhance chickpea and its byproduct “aquafaba” qualities and functional properties by high-pressure processing, especially for reducing antinutritional factors, soaking/hydration time, and improving functional properties of the associated proteins and carbohydrates	Wastewater from chickpeas and Dried grain	Aquafaba yield and protein content, color, turbidity, functional properties, tannin, phytic acid, hydrophobicity, emulsion particle size	Protein content: 0.5–1%	Emulsion properties were the maximum at 2:3 and cooking time of 60 min
		• Wastewater from chickpea canning			Foaming capacity was the highest (120%) at 2:3 cooked for 30 min
		◦ The proportion of grain/water was not mentioned	Method used to determine protein content:		The most stable foam was at 1:2 with 45 min cooking
			Bradford technique (Bradford 1976)		
		• Hydrated grain: Yes			
		◦ Proportion: no information			
		◦ Was the water discarded: no information			
		• Cooking:			
		◦ Pressure cooker for different times (15, 30, 45, and 60 min)			
		◦ Proportion (hydrated grain:water): 1:2; 1:4 and 2:3			
Meurer (2019) Brazil	Evaluate the effects caused by the use of ultrasound in the foaming and emulsifying functional properties of water cooking of chickpeas (aquafaba), to make it more efficient in replacing of egg in a food	• Hydrated grain: Yes	Foam capacity and stability	Carbohydrates: 2.05%	The results prove that the application of ultrasonic waves in aquafaba, at different times and intensities, favors its foaming capacity
		◦ Proportion: no information		Fat: 0.07%	
		Was the hydration water discarded? Yes	Method used to determine protein content: Method Kjeldhal, 036/IV (Instituto Adolfo Lutz, 2008)	Protein: 0.52%	
		• Cooking:			
		◦ Pressure cooker for 20 min			
		◦ Proportion (hydrated grain:water): 1:3			
Nguyen et al (2021) Vietnam and USA	Determine: (1) Effects of the different treatments on foaming capacity, foaming stability, hardness, and bubble size of foaming aquafaba and (2) Properties of cakes with the different treatments of chickpea cooking water as an application for eggless baking processes	• Hydrated grain: Yes	Foaming capacity and stability	Protein: 2.8%	The highest foaming ability was of the aquafaba solution with pH adjustment and table salt. with citric acid (pH of 4), table salt (3000 μg mL^−1^)
		◦ Proportion: no information-			
		◦ Was the hydration water discarded: Yes			
		• Cooking:			
		◦ Electric stove for 40 min			
		◦ Proportion (hydrated grain:water): 1:4			
Nguyệt (2019) Vietnam	Investigate the factors that affect the foam structure of chickpea cooking water for application in the processing of egg/milk/fat-free cold dessert products	• Hydrated grain: Yes	Foaming capacity and stability	No information	The result shows that the ratio of dry grains: water = 1:4 demonstrated high values of foam stability (%) and capacity (%)
		◦ Proportion: no information			
		◦ Was the hydration water discarded? no information			
		• Cooking:			
		◦ Boiling water for 45 min			
		◦ Proportion (hydrated grain): 1:3; 1:4 and 1:5			
Escadellas et al. (2022) France	Highlight the characteristics of aquafaba as a foaming food matrix and characterize its properties (rheology, foamability) with a view to the implementation of transformation processes	• Hydrated grain: -	Foaming properties	Composition considering wet basis:	Aquafaba has foaming properties as its foam has small, stable bubbles
		◦ Proportion: no information		Protein: 0.49%	
		◦ Was the water discarded? no information		Carbohydrates: 2.59%	
		• Cooking:		Hydrogen: 0.42%	
		◦ Proportion: no information		Nitrogen: 0.28%	
				Sulfur: < 0.05%	
Ricci (2018) Brazil	Physicochemical and rheological characterization of aquafaba analyzing the stability of the systems formed	• Hydrated grain:	Emulsions, foams, stability, and rheology	Composition considering wet basis:	All samples (emulsions and foams) from aquafaba and albumin showed phase separation; however, the separation remained stable after 5 h of observation
		◦ Proportion: 2:3		Protein: 1.57%	
		◦ Was the water discarded? Yes	Method used to determine protein content:	Carbohydrates: 2.03%	
		• Cooking:	Association of Official Analytical Chemists (AOAC) methods	Fat: 0.1%	
		◦ Proportion: no information			

*CCW* chickpea cooking water, *RSM* response surface methodology, *CWR* chickpea:boiling water ratio, *BCA* bicinchoninic acid

The evaluation of the nutritional composition of the aquafaba was performed in 58.8% (n = 10) of the included studies (Shim et al. 2018; Buhl et al. 2019a; Lafarga et al. 2019a; Meurer 2019; Nguyệt 2019; Alsalman et al. 2020a; Alsalman and Ramaswamy 2021a; Nguyen et al. 2021; Escadellas et al. 2022). Of them, tree (Escadellas et al. 2022) analyzed (Mustafa et al. 2018; Ricci 2018) (17.64%) composition on dry basis, and seven (41.17%) (Shim et al. 2018; Buhl et al. 2019a; Lafarga et al. 2019a; Meurer 2019; Nguyệt 2019; Alsalman et al. 2020a; Alsalman and Ramaswamy 2021a; Nguyen et al. 2021) on wet basis. Only 3 (17.64%) studies (Buhl et al. 2019a; Meurer 2019; Escadellas et al. 2022) assessed the proximate composition of the aquafaba. For comparison purposes, we converted the results from dry to wet bases. In general, carbohydrates ranged from 2.03 to 2.59%, and fat from 0.07 to 0.1. Considering protein, 47.05% of the studies performed the analysis, the minimum amount was 0.08%, and the maximum amount was 2.8% on a wet basis (Shim et al. 2018; Buhl et al. 2019a; Lafarga et al. 2019a; Meurer 2019; Nguyệt 2019; Alsalman et al. 2020a; Alsalman and Ramaswamy 2021a; Nguyen et al. 2021). Table 1 also presents the methods used to analyze the protein content in aquafaba, since it is the main nutrient involved in foam production.

About 53% of the studies informed the method used to analyze the protein content analysis. The most frequently (n = 5; 29.38%) used method was the Association of Official Analytical Chemists (AOAC 981.10), which is based on the Kjeldahl Method (Shim et al. 2018; He 2019; He et al. 2019; Landert et al. 2021). The second most used method was the Brad Ford technique (n = 2; 11.76%) (Alsalman 2020; Alsalman et al. 2020a). One study used the Kjeldahl Method without mentioning the correspondent AOAC method (Meurer 2019). Another study used the BCA method (Thermo Scientific™, USA) (Buhl et al. 2019a).

The studies had different ways of obtaining the aquafaba; 17.64% (Mustafa et al. 2018; Shim et al. 2018; Buhl et al. 2019a) used the wastewater from canned chickpeas, 17.64% (Alsalman 2020; Alsalman et al. 2020a; Landert et al. 2021) compared the wastewater of canned chickpeas, and home cooking of dry grains of chickpea and 58.82% (He 2019; He et al. 2019; Lafarga et al. 2019a; Meurer 2019; Nguyệt 2019; Aslan and Ertaş 2021; Editors et al. 2021; Nguyen et al. 2021; Escadellas et al. 2022) used home cooking of dry chickpea grains. Furthermore, the most used aquafaba formulation was with the grain previously hydrated with a proportion of 1:4 (dry chickpea/water) that was used in four studies (He et al. 2019; Lafarga et al. 2019a; Aslan and Ertaş 2021; Editors et al. 2021) as well as the wastewater from chickpea canning (Mustafa et al. 2018; Shim et al. 2018; Buhl et al. 2019a; Alsalman 2020; Alsalman et al. 2020a). Most studies did not mention whether water was discarded or not (Nguyệt 2019; Alsalman 2020; Alsalman et al. 2020a; Alsalman and Ramaswamy 2021a; Nguyen et al. 2021; Escadellas et al. 2022) and the majority used pressure-cooking for 30 min (He 2019; He et al. 2019; Meurer 2019; Alsalman 2020; Alsalman et al. 2020a; Alsalman and Ramaswamy 2021a; Landert et al. 2021).

Most studies (n = 12; 70.58%) used soaking in aquafaba production (He 2019; He et al. 2019; Lafarga et al. 2019a; Meurer 2019; Nguyệt 2019; Alsalman 2020; Alsalman et al. 2020a; Alsalman and Ramaswamy 2021a; Editors et al. 2021; Landert et al. 2021; Nguyen et al. 2021). However, only five (He 2019; He et al. 2019; Lafarga et al. 2019a; Editors et al. 2021) reported the proportion. Three of these used a proportion of 1:4 (chickpea:water), one study used a proportion of 1:3, and another used a proportion of 2:3. Only one study did not use the soaking technique (Aslan and Ertaş 2021) and the others used wastewater from chickpea canning.

As for the methods used for cooking, 32.29% of the studies (n = 6) (He 2019; He et al. 2019; Meurer 2019; Alsalman 2020; Alsalman et al. 2020a; Alsalman and Ramaswamy 2021a) used the pressure cooker. Nearly 20% of the studies (n = 3) used boiling water (Lafarga et al. 2019a; Nguyệt 2019; Editors et al. 2021). The cooking method employed in the other eight studies (47.05%) (Mustafa et al. 2018; Shim et al. 2018; Buhl et al. 2019a; Aslan and Ertaş 2021; Landert et al. 2021; Nguyen et al. 2021; Escadellas et al. 2022) was not mentioned.

### Risk of bias (RB)

The studies are heterogeneous, but the majority, 88.23%, had a low risk of bias, 5.88% had a moderate risk of bias, and 5.88% presented a high risk of bias (Table 2). All studies answered the main question.

**Table 2:** Summarized risk of bias assessment

Author (year)	Risk of bias	Risk (%)
He et al. (2019)	Low	100
Buhl et al. (2019a)	Low	100
Aslan and Ertaş (2021)	Low	100
Mustafa et al. (2018)	Low	100
Alsalman et al. (2020a)	Low	100
Landert et al. (2021)	Low	100
Alsalman and Ramaswamy (2021a)	Low	100
Lafarga et al. (2019a)	Low	100
Shim et al. (2018)	Moderate	50
Nguyet & Quoc & Buu (Editors et al. 2021)	Low	100
He (2019)	Low	100
Alsalman (2020)	Low	100
Meurer (2019)	Low	100
Nguyen et al. (2021)	Low	100
Nguyệt (Nguyệt 2019)	Low	100
Escadellas et al. (2022)	High	16.6
Ricci (2018)	Low	100

## Discussion

### Studies characteristics

The number of vegetarianism and veganism followers has grown and, consequently, the search for products that can replace food and ingredients of animal origin (Révillion et al. 2020). This growth tendency is mainly found in high-income countries (Leitzmann 2014; Ginsberg 2018). About 10% of the total population declares themselves vegetarians in countries like Australia, New Zealand, Israel, and Sweden. In India, given the prominent religion, one-third of the population is vegetarian (Iguacel et al. 2021). Therefore, following the trend of the recent growth of the vegetarian movement, the studies included in this systematic review topic were conducted recently.

In general, most of the studies were performed in Canada (n = 7; 41.17%), followed by Vietnam (n = 3; 17.64%), Brazil (n = 3; 17.64%) and China (n = 2; 11.76%). Among the countries included in the studies evaluated in this review, data suggest that vegetarianism is most prevalent in Brazil (14% of the population) (Sociedade Brasileira Vegetariana—SBV 2018), followed by Canada (12.2%) (Cudmore 2021), Vietnam and Denmark (10%) (Ăn Chay—XU HƯỚNG MỚI CỦA LỐI SỐNG HIỆN ĐẠI (PHẦN 1)—Nhịp Cầu Thế Giới Online 2011, Motrøen 2019), France (5.2%) (Avelin 2019), China and USA (5%) (Chinese vegetarian: China’s vegetarian population touches 50 million: Report—Times of India 2014, Tapper 2021) and Spain (1.4%) (EFEAGRO 2021). Therefore, among the countries included in this review, the countries that have most of the studies on this topic were the ones with the highest prevalence of vegetarianism, except for Denmark.

### Aquafaba production

**Fig. 2:**
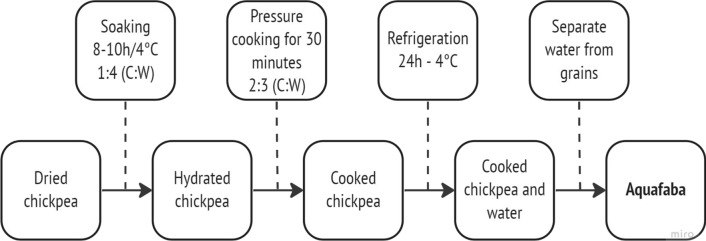
Homemade chickpea aquafaba production flowchart

Aquafaba can be obtained using two primary sources: homemade cooking of chickpeas wastewater or separating the viscous liquid from canned chickpeas. These different primary sources interfere with the foam capacity and stability properties because of the individual characteristics of the grain, the type of implemented storage (if refrigerated or not) and time of storage, cooking time and temperature, and the use of pressure (Mustafa et al. 2018; He et al. 2019). The homemade process of aquafaba production (Fig. 2) in most of the studies was: the dried chickpeas were soaked for 8–10 h at 4 °C on a proportion of 1:4 (chickpea:water) (He 2019; He et al. 2019; Editors et al. 2021). After that, the water was discarded, and the hydrated grains went into pressure cooking for 30 min on a proportion of 2:3 (hydrated chickpea:water) (Alsalman 2020; Alsalman et al. 2020a; Landert et al. 2021). Subsequently, the cooked chickpeas with the cooking water were refrigerated for 24 h at 4 °C. By the end, the wastewater (aquafaba) was separated from the grains. None of the studies described exactly the protocol displayed in Fig. 2; however, this protocol was constructed based on the most frequent processes in the evaluated studies.

### Soaking and cooking processes

Regarding the soaking step, 94.11% of the studies (Mustafa et al. 2018; Shim et al. 2018; Buhl et al. 2019a; He 2019; He et al. 2019; Lafarga et al. 2019a; Meurer 2019; Nguyệt 2019; Alsalman 2020; Alsalman et al. 2020a; Alsalman and Ramaswamy 2021a; Editors et al. 2021; Landert et al. 2021; Nguyen et al. 2021; Escadellas et al. 2022) previously hydrated the chickpea grains, but only 29,41% of them (He 2019; He et al. 2019; Lafarga et al. 2019a; Editors et al. 2021) mentioned the proportion of water and chickpeas. The hydration capacity of the grain during soaking is generally related to the physical properties of the grain. Thus, different effects on aquafaba may be noted. A study showed that it was impossible to obtain foam when chickpeas were not soaked (Landert et al. 2021). Soaking is a common process among pulses since the mechanical process of adding water before the cooking process and letting the grains rest underwater for a minimum of eight hours might improve digestion by reducing antinutritional phytates and oxalates while also softening the soaked grain (Fernandes et al. 2010). The hydration promoted by soaking results in the swelling of the seed’s cotyledons, making the seed coat crack more permeable (Fernandes et al. 2010; Shafaei et al. 2016). At the same time, the introduced water partially hydrates starch molecules inside the matrix in a rheological phenomenon called gelation (Morris 1990). Combining these processes reduces the cooking time and regulates the chemical diffusion into the cooking water (aquafaba). Some studies mentioned that after soaking and cooking, the total amounts of sugar, oligosaccharides (raffinose, stachyose, verbascose), and protein in chickpeas decreased, given that a part of these compounds was diffused into the cooking water (He et al. 2019, 2021; Alsalman 2020; Alsalman et al. 2020a). Among the compounds diffused into the cooking water, proteins related to aquafaba formation might be more prominent than seeds that skipped the soaking process. Also, it is important to note that most studies discarded the water residual from soaking because of antinutritional compounds (He et al. 2019, 2021; Alsalman 2020; Alsalman et al. 2020a).

The ratio 1:4 (hydrated grains:water) was the most used proportion (n = 5; 29.41%) in the cooking step (Nguyệt 2019; Alsalman 2020; Alsalman et al. 2020a; Editors et al. 2021; Nguyen et al. 2021). However, regarding the technological characteristics of aquafaba, it was not the one with the best results. Therefore, we used the proportion with the best results mentioned in the studies to construct the flowchart (Fig. 2). Although the 2:3 ratio was only tested in three studies, this ratio showed better characteristics, emulsion properties, and foaming capacity in studies that compared it with the 1:4 proportion (Alsalman 2020; Alsalman et al. 2020a). Only one study showed that on the proportion of 1:4 with the pH adjustment with the addition of table salt and citric acid (pH of 4), table salt (3.000 μg mL^−1^) performed the highest foaming ability during the whipping (Nguyen et al. 2021); however, this study did not compare with the aquafaba using the proportion of 2:3 on cooking.

According to a study (Vidal-Valverde et al. 1993), the total sugar content of chickpeas was significantly reduced after boiling in water (32% of reduction). Non-galactoside sugars (fructose and sucrose) decreased slightly more than galactoside sugars (38 and 50% decrease, respectively) (Frias et al. 2000). The solubilization of carbohydrates in water can partly explain these carbohydrate losses during the soaking and cooking process. However, because other water-soluble nutrients were also eliminated, the soluble sugar losses are significantly higher than the percentages shown (Vidal-Valverde et al. 1993). In that case, the aquafaba produced through boiling water could have good foaming stability, given that the solubilization of carbohydrates in the cooking water positively influences aquafaba’s foam stability (Vidal-Valverde et al. 1993).

Protein denaturation causes structural changes, which cause protein modifications. These modifications either increase molecule size through aggregation (lower solubility) or decrease it through breakdown into smaller compounds (increased solubility) (Alsalman and Ramaswamy 2021a). As for high-pressure cooking, rupturing non-covalent connections between protein molecules or forming new intermolecular links (such as hydrogen bonds and hydrophobic interactions) promotes protein aggregation]. Due to changes in solvation volume, where non-covalent bonds are ruptured and reorganized with solvent molecules, pressure cooking can also increase protein volume (Alsalman and Ramaswamy 2021a).

The different proportions of water seem to affect the technological properties of the aquafaba. Lower quantities of water make the chickpeas soft and crumbly, allowing the starch granules to easily disperse in the cooking water and break the foam membranes, thus lowering the foaming capacity and stability (Editors et al. 2021). Excess water also impairs foam formation by excessive solubilization of starch and protein, thus reducing this compound's concentration (Landert et al. 2021). Regarding refrigeration, according to Landert et al. (2021), this practice, if carried out for 24 h after cooking the beans, significantly improves foam formation and stability. Probably, the cooling time favored chemical reactions such as the starch gelation and extravasation of proteins from the cooked grain to the wastewater. Thus, it results in a greater amount of gelated starch and solubilized proteins, favoring a more technologically stable aquafaba (Landert et al. 2021).

### Canned chickpea

The studies that investigated aquafaba from the wastewater of canned chickpeas (Mustafa et al. 2018; Shim et al. 2018; Buhl et al. 2019a) showed that the manufacturers that produced the commercial brands have different genetic chickpea cultivars causing changes in the nutrition composition of the aquafaba, as well as foaming and emulsifying properties (Mustafa et al. 2018; He et al. 2019). In general, the proximate composition of the utilized chickpeas did not influence the stability of aquafaba; nevertheless, it seems that grains with higher amounts of dry matter displayed better emulsion proprieties, resulting in better results for aquafaba (Mustafa et al. 2018; He et al. 2019). Nevertheless, the dry matter content relies mainly on the chickpeas’ genotype, given that the aquafaba produced by the *“CDC Leader”* genotype presented the highest amount of dry matter and, subsequently, the most adequate aquafaba (He et al. 2019).

In addition, some commercial brands include food additives, such as salt, and preservatives like disodium ethylene diamine tetra acetic acid (EDTA), which might suppress viscosity and foam stability by increasing the molecular weight of the formulation (Mustafa et al. 2018; Shim et al. 2018). In this manner, the aquafaba from brands that had no addition of salt or additives produced more dense foam with greater capacity and stability (Mustafa et al. 2018; Shim et al. 2018; Eren et al. 2021). Some food additives, such as citric acid (provided by lime and lemon juices) can be implemented to enhance the stability of aquafaba. A study showed that lowering the pH and decreasing the chickpea:water ratio of manufactured aquafaba improved both foaming and emulsifying capacity of aquafaba (Lafarga et al. 2019b). Other pH-lowering strategies are also successfully improving aquafaba’s technological capacities, adding cream of tartar, another acidic ingredient, increased foam and stability (Wong 2020).

Some other studies compared the aquafaba with dried beans (homemade) and canned chickpeas. They concluded that the proportion of 2:3 of homemade aquafaba had the best results, forming foam more quickly and with high stability. The aquafaba from canned chickpeas has a higher foam volume and lower emulsion properties than the homemade cooking conditions, possibly because of the utilized chickpea cultivars; however, these studies did not describe the used chickpea cultivar (Alsalman 2020; Alsalman et al. 2020a; Landert et al. 2021).

### Chemical composition

Aquafaba is mainly composed of carbohydrates and protein, but protein is the most evaluated compound in the studies due to its foaming properties (Shim et al. 2018; Buhl et al. 2019a; Alsalman et al. 2020a; Landert et al. 2021; Escadellas et al. 2022). Proteins may present hydrophilic amino acids interacting with water, whereas the hydrophobic amino acids stabilize interactions with the gaseous phase. In this sense, aquafaba foaming capacity strongly correlates with protein content (He 2019). A lack of a standard for protein measurements was observed among studies. Also, some studies showed the content on a dry basis and others on a wet basis. Studies (Shim et al. 2018; Landert et al. 2021) used the same technique for analyzing proteins (AOAC). The only difference in the method is that one of the articles multiplied the nitrogen content by 6.25 and the other by 5.75, probably because the majority of the studies used the general 6.25 factor, and the study that used 5.75 was specific for chickpeas. Despite this, the studies showed different results regarding protein, 1.7% protein for canned chickpeas and 3% protein for homemade chickpeas. The differences in protein content also occur because of the different cooking methods. Boiling can change nutrients’ concentrations. There may be solubilization of proteins or even a higher concentration (Lafarga et al. 2019a; Landert et al. 2021).

Only two studies evaluated fat content in chickpea aquafaba, 0.07% (Meurer 2019) and 0.1% on a wet basis [42]. This data is important, since fat can influence the foaming capacity of aquafaba. The presence of unsaturated fatty acids reduces the volume and stability of the foam, and chickpea cultivars may contain 2.70–6.50% of fat. It is an important source of unsaturated fatty acids (Behera et al. 2014; Kaur and Prasad 2021). And these two that evaluated fat had lower or higher foaming capacity.

In which regards aquafaba use as a functional ingredient, this plant-based foam was already implemented as a substitute for egg whites in vegan mayonnaises, and in eggless sponge cakes, providing satisfactory results regarding nutritional value, technological and sensory aspects (Mustafa et al. 2018b, Buhl et al*.* 2019a). Another study analyzed physicochemical and microbiological indicators of aquafaba, and proved that under refrigeration or in the form of dehydrated powder, aquafaba could be used as a viable product, reinforcing its economic value (Ahmed et al. 2021).

## Conclusion

This study aimed to evaluate different methods for obtaining aquafaba (soaking type, time, proportion of water or using canned chickpea; use of soaking water; cooking type, time, proportion of water; storage temperature and time; beating time) and compare their nutritional and technological characteristics. The results showed the following steps to prepare aquafaba: soaking for 8–10 h at 4 °C at the proportion of 1:4 (chickpea:water), pressure cooking for 30 min in the proportion of 2:3 (chickpea:water), and refrigerating 24 h/4 °C. Most of the studies used soaking in water as a strategy to home-cook chickpeas, improving the diffusion of compounds to the water in the cooking process. The composition of chickpeas did not alter the quality of the aquafaba produced; however, species with higher concentrations of dry matter produced better foam. According to the studies, there was also an indication that aquafaba from wastewater canned chickpeas produced by the *CDC Leader* chickpeas genotype presented better results regarding foam formation, emulsion capacity, and stability compared to homemade aquafaba. Also, canned chickpeas without added salt or EDTA produced aquafaba with better technological characteristics.

### Supplementary Information

Below is the link to the electronic supplementary material.

Supplementary file1 (DOCX 112 KB)

## Data Availability

The work has no associated data.

## Data availability

This work has no associated data.
