# Supplementary material for: Chickpea aquafaba: a systematic review of the different processes for obtaining and their nutritional and technological characteristics
Source: J Food Sci Technol. 2024 Jan 26;61(8):1439–56. doi: 10.1007/s13197-023-05920-y (PMC11219635; doi:10.1007/s13197-023-05920-y)
Supplement: Supplementary file 1 — Supplementary file1 (DOCX 112 KB) [file 13197_2023_5920_MOESM1_ESM.docx]

**Table S1.** Full-text excluded articles and reasons.

| **Author (year)** | **Reference** | **Exclusion reason** |
| --- | --- | --- |
| He et al. (2021) | (He *et al.* 2021) | 2 |
| Alsalman & Ramaswamy (2021) | (Alsalman and Ramaswamy 2021) | 3 |
| Saget et al (2021) | (Saget *et al.* 2021) | 2 |
| Raikos & Hayes & Ni (2020) | (Raikos, Hayes, and Ni 2020) | 2 |
| Muhialdin et al. (2020) | (Muhialdin *et al.* 2021) | 2 |
| Mustafa & Reaney (2020) | (Mustafa and Reaney 2020) | 1 |
| Donatus et al. (2020) | (Donatus *et al.* 2020) | 4 |
| Setarehnejad & Hall (2021) | (Setarehnejad and Hall 2021) | 1 |
| Mazur et al, (2021) | (Mazur *et al.* 2021) | 1 |
| Aslan & Ertas (2020) | (Aslan and Ertaş 2018) | 2 |
| Armaforte & Hopper & Stevenson (2021) | (Armaforte *et al.* 2021) | 4 |
| Silva (2020) | (Silva *et al.* 2020) | 2 and 4 |
| Yuccer (2021) | (Yüceer 2021) | 1 |
| Raikos et al (2020) | (Raikos, Hayes, Agriopoulou, *et al.* 2020) | 3 |
| Shim & Hayes (2020) | (Shim and Reaney 2020) | 1 |
| Kim et al. (2021) | (Young Kim *et al.* 2021) | 3 |
| Anwar et al. (2019) | (Anwar *et al.* 2019) | 1 |
| Santos et al. (2019) | (Santos *et al.* 2021) | 1 |
| Божко т et al. (2020) | (Божко *et al.* 2020) | 4 |
| Макарова et al. (2021) | (Макарова Н.В. *et al.* 2021) | 4 |
| Березовикова (2018) | (Березовикова *et al.* 2018) | 1 |
| Lebrun (2020) | (Lebrun 2020) | 2 |
| Sugiarto (2019) | (Sugiato 2019) | 4 |
| Brennan et al. (2022) | (Brennan *et al.* 2022) | 2 |
| Shim et al. (2020) | (Shim *et al.* 2018) | 3 |
| Elizabeth & Lissette (2020) | (Elizabeth and Lissette 2020) | 2 |

Legend – Exclusion criteria: 1 - comments, letters, conference, review, abstracts, papers, and books (n= 8); 2 -studies that do not evaluate the properties of aquafaba, but try to include it in the formulation of a product (n= 9); 3 - it does not evaluate the properties, but improves aquafaba through treatments (n=4); 4 - studies evaluating aquafaba from different pulses (n=5);

**References**

1. He, Y.; Purdy, S.K.; Tse, T.J.; Tar’an, B.; Meda, V.; Reaney, M.J.T.; Mustafa, R. Standardization of Aquafaba Production and Application in Vegan Mayonnaise Analogs. *Foods 2021, Vol. 10, Page 1978* **2021**, *10*, 1978, doi:10.3390/FOODS10091978.

2. Alsalman, F.B.; Ramaswamy, H.S. Evaluation of Changes in Protein Quality of High-Pressure Treated Aqueous Aquafaba. *Molecules* **2021**, *26*, doi:10.3390/MOLECULES26010234.

3. Saget, S.; Costa, M.; Styles, D.; Williams, M. Does Circular Reuse of Chickpea Cooking Water to Produce Vegan Mayonnaise Reduce Environmental Impact Compared with Egg Mayonnaise? *Sustainability 2021, Vol. 13, Page 4726* **2021**, *13*, 4726, doi:10.3390/SU13094726.

4. Raikos, V.; Hayes, H.; Ni, H. Aquafaba from Commercially Canned Chickpeas as Potential Egg Replacer for the Development of Vegan Mayonnaise: Recipe Optimisation and Storage Stability. *International Journal of Food Science & Technology* **2020**, *55*, 1935–1942, doi:10.1111/IJFS.14427.

5. Muhialdin, B.J.; Mohammed, N.K.; Cheok, H.J.; Farouk, A.E.A.; Meor Hussin, A.S. Reducing Microbial Contamination Risk and Improving Physical Properties of Plant-Based Mayonnaise Produced Using Chickpea Aquafaba. *International Food Research Journal* **2021**, *28*, 457–553.

6. Mustafa, R.; Reaney, M.J.T. Aquafaba, from Food Waste to a Value-Added Product. *Food Wastes and By‐products* **2020**, 93–126, doi:10.1002/9781119534167.CH4.

7. Donatus, F.; Sintang, M.D.; Julmohammad, N.; Ab Wahab, N. Potential Application of Unconsumed Liquid from Commercial Canned Food Products in Fabrication and Characterisation of Non-Dairy Edible Foam - UMS INSTITUTIONAL REPOSITORY Available online: https://eprints.ums.edu.my/id/eprint/32237/ (accessed on 9 May 2022).

8. Setarehnejad, A.; Hall, N. An Investigation in the Characteristics and Properties of Aquafaba and Its Use in Large Scale Manufacturing. *J Acad Nutr Diet* **2021**, *121*, A17, doi:10.1016/J.JAND.2021.06.030.

9. Mazur, M.; Sandulachi, E.; Patras, A.; Ghendov-Mosanu, A. USE OF CHICKPEAS AQUAFABA IN THE TECHNOLOGY OF MANUFACTURING VEGETAL SPONGE CAKE. In Proceedings of the International Conference INTELLIGENT VALORISATION OF AGRO-INDUSTRIAL WASTES; Chisinau, 2021; p. 48.

10. Aslan, M.; Ertaş, N. Harran Tarım ve Gıda Bilimleri Derg. **2018**, *2020*, 1–8, doi:10.29050/harranziraat.569397.

11. Armaforte, E.; Hopper, L.; Stevenson, G. Preliminary Investigation on the Effect of Proteins of Different Leguminous Species (Cicer Arietinum, Vicia Faba and Lens Culinarius) on the Texture and Sensory Properties of Egg-Free Mayonnaise. *LWT* **2021**, *136*, 110341, doi:10.1016/J.LWT.2020.110341.

12. Silva, P.G.; De, D.; Medianeira, M. SUBSTITUIÇÃO DE OVOS EM BOLO SEM GLÚTEN POR PREPARADO VEGETAL: DESENVOLVIMENTO, CARACTERIZAÇÃO E APLICAÇÃO TECNOLÓGICA, Universidade Tecnológica Federal do Paraná: Medianeira, 2020.

13. Yüceer, M. Yumurta Alternatifleri ve Yumurta İkamesi Olarak Kullanım İmkanları - Egg Replacers and Use as Egg Substitute | Muhammed Yüceer - Academia.Edu Available online: https://www.academia.edu/61262852/Yumurta_Alternatifleri_ve_Yumurta_%C4%B0kamesi_olarak_Kullan%C4%B1m_%C4%B0mkanlar%C4%B1_Egg_Replacers_and_Use_as_Egg_Substitute (accessed on 9 May 2022).

14. Raikos, V.; Hayes, H.E.; Agriopoulou, S.; Varzakas, T. Proteomic Dataset of Aquafaba from Canned Chickpea (Cicer Arietinum L.) Broth. *Current Topics in Peptide & Protein Research* **2020**, *21*, 61–68.

15. Shim, Y.Y.; Reaney, M.J.T. What Is the New Vegan Secret Food, Aquafaba? Available online: http://kren.rinfo.kr/search/detail/DetailView.do?p_mat_type=1a0202e37d52c72d&control_no=ba724533d077769bc85d2949c297615a&keyword= (accessed on 9 May 2022).

16. Young Kim, H.; Gyun Choi, S.; Jin Kang, S.; Sun Shin, W.; Young Shim, Y.; Reaney, M.J.; Hye Kim, J.; Youl Cho, J.; Soo Hong, W.; Hong, W.-S. Awareness of Vegetarian-Based Food (Aquafaba) and Vegetarian Restaurant According to the Food Consumption Value of Vegetarians. *Journal of the Korean Society of Food Culture* **2021**, *36*, 430–440, doi:10.7318/KJFC/2021.36.5.430.

17. Anwar, A.; Khalil, H.; Tayyab, S.; Ali, R. UTILIZATION OF AQUAFABA AS AN EGG REPLACER FOR EGG INTOLERANT IN THE PRODUCTION OF VEGAN MAYONNAISE | Advanced Food and Nutritional Sciences. *Advanced Food and Nutritional Sciences* **2019**, *4*, 29–30.

18. Santos, C.A.C.; Fernández, E.A.M.; Ribeiro, V.M.S.; Simões, R.O.; Stamford, T.L. Água de Cozimento Do Grão-de-Bico e as Suas Propriedades Tecnológicas: Uma Revisão. *Avanços em Ciência e Tecnologia de Alimentos* **2021**, *3*, 1–24, doi:10.37885/210203229.

19. Божко, С.Д.; Ершова, Т.А.; Чернышева, А.Н.; Черногор, А.М. Legumes Are a Promising Raw Material for the Food Industry. *Технологии пищевой и перерабатывающей промышленности АПК–продукты здорового питания* **2020**, *2*, 59–64.

20. Макарова Н.В.; Воронина М.С.; Гуляева А.Н.; Нистерюк Д.И.; Шляпникова Э.Н. АНАЛИЗ СОДЕРЖАНИЯ СУХИХ ВЕЩЕСТВ, БЕЛКА И ТИТРУЕМОЙ КИСЛОТНОСТИ В ОТВАРАХ БОБОВЫХ. *Food Technology* **2021**, *3*, 1.

21. Березовикова, И.П.; Тюрина, В.В.; Балде, О.Р. Новые Продукты Здорового Питания На Основе Нута. In Proceedings of the Дни науки; 2018; pp. 20–25.

22. Lebrun, D. Conception d’une Mousse Au Chocolat Végétale Instantanée à Partir d’aquafaba, Université de Liège: Liege, 2020.

23. Sugiato, Y. EFFECT OF CHICKPEAS (Cicer Arietinum L.) AND SOYBEAN (Glycine Max L.) AQUAFABA AS EGG WHITE SUBSTITUES ON THE PHYSICOCHEMICAL AND ORGANOLEPTIC PROPERTIES OF ROYAL ICING DURING STORAGE - Unika Repository, UNIVERSITAS KATOLIK SOEGIJAPRANATA: SEMARANG, 2019.

24. Brennan, C.; Mustafa, R.; Boukid, F.; Gagaoua, M. Vegan Egg: A Future-Proof Food Ingredient? *Foods 2022, Vol. 11, Page 161* **2022**, *11*, 161, doi:10.3390/FOODS11020161.

25. Shim, Y.Y.; Mustafa, R.; Shen, J.; Ratanapariyanuch, K.; Reaney, M.J.T. Composition and Properties of Aquafaba: Water Recovered from Commercially Canned Chickpeas. *J Vis Exp* **2018**, *2018*, doi:10.3791/56305.

26. Elizabeth, B.H.F.; Lissette, E.A.M. Anexo VI: Certificado Del Docente Tutor Del Trabajo de Titulación Guayaquil, 4 de Octubre de 2020, Universidad de Guayaqu: Guayaquil, 2020.

**Table S2.** Indexers used to select publications that jointly or separately address words related to aquafaba and its properties.

| **LILACS** | ("Aquafaba" ) **AND** ("chickpea" OR "chickpeas") **AND** (“emulsion”);  ("Aquafaba" ) **AND** ("chickpea" OR"chickpeas") **AND** (“foam”); ("Aquafaba" ) **AND** ("chickpea" OR "chickpeas") **AND** (“functional properties” OR “functional property”);  ("Aquafaba" ) **AND** ("chickpea" OR "chickpeas") **AND** (“stability”); ("Aquafaba" ) **AND** ("chickpea" OR "chickpeas") **AND** (“nutritional properties” OR “nutritional property”);  ("Aquafaba") **AND** ("chickpea" OR "chickpeas") **AND** (“Nutritional quality” OR “Nutritional Qualities”); |
| --- | --- |
| **Pubmed** | ("Aquafaba" ) **AND** ("chickpea" OR "chickpeas") **AND** (“emulsion”);  ("Aquafaba" ) **AND** ("chickpea" OR"chickpeas") **AND** (“foam”); ("Aquafaba" ) **AND** ("chickpea" OR "chickpeas") **AND** (“functional properties” OR “functional property”);  ("Aquafaba" ) **AND** ("chickpea" OR "chickpeas") **AND** (“stability”); ("Aquafaba" ) **AND** ("chickpea" OR "chickpeas") **AND** (“nutritional properties” OR “nutritional property”);  ("Aquafaba") **AND** ("chickpea" OR "chickpeas") **AND** (“Nutritional quality” OR “Nutritional Qualities”); |
| **ProQuest** | ("Aquafaba" ) **AND** ("chickpea" OR "chickpeas") **AND** (“emulsion”);  ("Aquafaba" ) **AND** ("chickpea" OR"chickpeas") **AND** (“foam”); ("Aquafaba" ) **AND** ("chickpea" OR "chickpeas") **AND** (“functional properties” OR “functional property”);  ("Aquafaba" ) **AND** ("chickpea" OR "chickpeas") **AND** (“stability”); ("Aquafaba" ) **AND** ("chickpea" OR "chickpeas") **AND** (“nutritional properties” OR “nutritional property”);  ("Aquafaba") **AND** ("chickpea" OR "chickpeas") **AND** (“Nutritional quality” OR “Nutritional Qualities”); |
| **SCOPUS** | ("Aquafaba" ) **AND** ("chickpea" OR "chickpeas") **AND** (“emulsion”);  ("Aquafaba" ) **AND** ("chickpea" OR"chickpeas") **AND** (“foam”); ("Aquafaba" ) **AND** ("chickpea" OR "chickpeas") **AND** (“functional properties” OR “functional property”);  ("Aquafaba" ) **AND** ("chickpea" OR "chickpeas") **AND** (“stability”); ("Aquafaba" ) **AND** ("chickpea" OR "chickpeas") **AND** (“nutritional properties” OR “nutritional property”);  ("Aquafaba") **AND** ("chickpea" OR "chickpeas") **AND** (“Nutritional quality” OR “Nutritional Qualities”); |
| **Embase** | ("Aquafaba" ) **AND** ("chickpea" OR "chickpeas") **AND** (“emulsion”);  ("Aquafaba" ) **AND** ("chickpea" OR"chickpeas") **AND** (“foam”); ("Aquafaba" ) **AND** ("chickpea" OR "chickpeas") **AND** (“functional properties” OR “functional property”);  ("Aquafaba" ) **AND** ("chickpea" OR "chickpeas") **AND** (“stability”); ("Aquafaba" ) **AND** ("chickpea" OR "chickpeas") **AND** (“nutritional properties” OR “nutritional property”);  ("Aquafaba") **AND** ("chickpea" OR "chickpeas") **AND** (“Nutritional quality” OR “Nutritional Qualities”); |
| **Web of Science** | TS = ("Aquafaba" ) **AND** TS = ("chickpea" OR "chickpeas") **AND** TS= (“emulsion”);  TS = ("Aquafaba" ) **AND** TS= ("chickpea" OR "chickpeas") **AND** TS= (“foam”);  TS= ("Aquafaba" ) **AND** TS= ("chickpea" OR "chickpeas") **AND** TS= (“functional properties” OR “functional property”);  TS= ("Aquafaba" ) **AND** TS= ("chickpea" OR "chickpeas") **AND** TS= (“stability”);  TS= ("Aquafaba" ) **AND** TS= ("chickpea" OR "chickpeas") **AND** TS= (“nutritional properties” OR “nutritional property”);  TS= ("Aquafaba" ) **AND** TS= ("chickpea" OR "chickpeas") **AND** TS= (“Nutritional quality” OR “Nutritional Qualities’); |
| **Google Scholar** | ("Aquafaba" ) **AND** ("chickpea" OR "chickpeas") **AND** (“emulsion”);  ("Aquafaba" ) **AND** ("chickpea" OR"chickpeas") **AND** (“foam”); ("Aquafaba" ) **AND** ("chickpea" OR "chickpeas") **AND** (“functional properties” OR “functional property”);  ("Aquafaba" ) **AND** ("chickpea" OR "chickpeas") **AND** (“stability”); ("Aquafaba" ) **AND** ("chickpea" OR "chickpeas") **AND** (“nutritional properties” OR “nutritional property”);  ("Aquafaba") **AND** ("chickpea" OR "chickpeas") **AND** (“Nutritional quality” OR “Nutritional Qualities”); |

**Table S3.** Quality criteria of the studies selected for the systematic review of chickpea aquafaba.

| Reference | Was the Study design appropriate? | | Was the statistical analysis adequate to the objective of the study? | Were objective, standard criteria used for measurement of the condition? | Did the results answer the main question? | Were strategies to deal with confounding factors stated? | Were the outcomes measured in a valid and reliable way? | Risk Percentage |
| --- | --- | --- | --- | --- | --- | --- | --- | --- |
| He et al. (2019) (He *et al.* 2019) | | Y | Y | Y | Y | N/A | Y | 100% |
| Buhl & Christensen & Hammershøj (2019) [6] | | Y | Y | Y | Y | N/A | Y | 100% |
| Aslan & Ertas (2020) [13] | | Y | Y | Y | Y | N/A | Y | 100% |
| Mustafa et al. (2018) [9] | | Y | Y | Y | Y | N/A | Y | 100% |
| Alsalman et al. (2020) [2] | | Y | Y | Y | Y | N/A | Y | 100% |
| Landert & Zaminelli & Capitani [11] | | Y | Y | Y | Y | N/A | Y | 100% |
| Alsalman & Ramaswamy (2020) [1] | | Y | Y | Y | Y | N/A | Y | 100% |
| Lafarga et al. (2019) [12] | | Y | Y | Y | Y | N/A | Y | 100% |
| Shim et al. (2018) [8] | | N | Y | Y | N/A | N/A | Y | 50% |
| Nguyet & Quoc & Buu (2021) [14] | | Y | Y | Y | Y | N/A | Y | 100% |
| He (2019) [15] | | Y | Y | Y | Y | N/A | Y | 100% |
| Alsalman (2020) [16] | | Y | Y | Y | Y | N/A | Y | 100% |
| Meurer (2019) [7] | | Y | Y | Y | Y | N/A | Y | 100% |
| Nguyen et al (2021) [17] | | Y | Y | Y | Y | N/A | Y | 100% |
| Nguyêt (2019) (Nguyệt 2019) | | Y | Y | Y | Y | Y | Y | 100% |
| Escadellas et al. (2019)[21] | | N | N | Y | N/A | N/A | N/A | 16,6% |
| Ricci (2018) [42] | | Y | Y | Y | Y | N/A | Y | 100% |
